# From metabolic antagonism to homeostatic restoration: rewiring hepatic stellate cell bioenergetics for liver fibrosis reversal

**DOI:** 10.3389/fmed.2026.1912158

**Published:** 2026-09-07

**Authors:** Xinyu Lu, Zilong Li

**Affiliations:** Department of Endocrinology, Jinan Central Hospital Affiliated to Shandong First Medical University, Jinan, China

**Keywords:** hepatic stellate cells (HSC), liver fibrosis, metabolic reprogramming, amino acid metabolism, lipid metabolism, glycolysis

## Abstract

Emerging evidence has redefined metabolic reprogramming in hepatic stellate cells (HSCs) from a mere energy-supporting process to an active driver of liver fibrosis. Unlike conventional descriptions that focus on isolated pathway alterations, recent advances show that activated HSCs coordinate a multi-layered metabolic remodeling involving glucose, lipid, and amino acid networks. This review highlights three paradigm-shifting insights. First, glycolytically derived lactate not only serves as a metabolic byproduct but also acts as an epigenetic signal through histone lactylation, Which may contribute to the maintenance of fibrogenic gene expression. Second, metabolic crosstalk—exemplified by GLUT1-containing exosomes released from activated HSCs that reprogram quiescent neighboring cells—has been reported to mediate intercellular propagation of the fibrotic phenotype. Third, HSC activation is characterized by stage-specific metabolic vulnerabilities: early lipophagy supports fatty acid oxidation, whereas fully activated HSCs transition to a unique “dual hypermetabolic” state featuring concurrent glycolysis and tricarboxylic acid cycle engagement. Translational efforts have moved beyond single-pathway inhibition toward restoring metabolic homeostasis, with clinical-stage agents such as EVT0185 (a dual ACLY/ACSS2 inhibitor), VDR agonists, and ROCK2 inhibitors showing early promise. We propose that future anti-fibrotic strategies should prioritize stage-specific metabolic signatures, dynamic metabolomics-guided trial design, and the development of highly selective metabolic modulators that reverse, rather than merely block, HSCs activation. A metabolism-centric approach may provide a promising therapeutic direction for the management of chronic liver disease.

## 1 Introduction

Liver cirrhosis is characterized by diffuse liver fibrosis and structural disorganization resulting from the progression of various chronic liver diseases. The disease burden associated with cirrhosis is increasingly severe and has become a major focus in global public health. According to statistics, there are currently 112 million patients with compensated cirrhosis and 10.6 million with decompensated cirrhosis worldwide. Each year, more than 1.32 million deaths are attributed to cirrhosis-related diseases, accounting for approximately 2.4% of total global deaths. Among the approximately 2 million annual deaths from liver diseases worldwide, 1 million are caused by cirrhosis, and the other 1 million are caused by viral hepatitis and hepatocellular carcinoma (HCC). Currently, cirrhosis is the 11th most common cause of death globally, and among people aged 45–64 years, it ranks third (tied with liver malignancy) (1). The situation is particularly severe in China, a country with a high burden of liver disease, where 50% of global deaths from cirrhosis occur (2). The total number of patients with cirrhosis in China exceeds 7 million, with approximately 800,000 new cases each year. Notably, 37% of these patients are under 45 years of age, showing a clear trend toward a younger age at onset (3).

**Figure 1.**
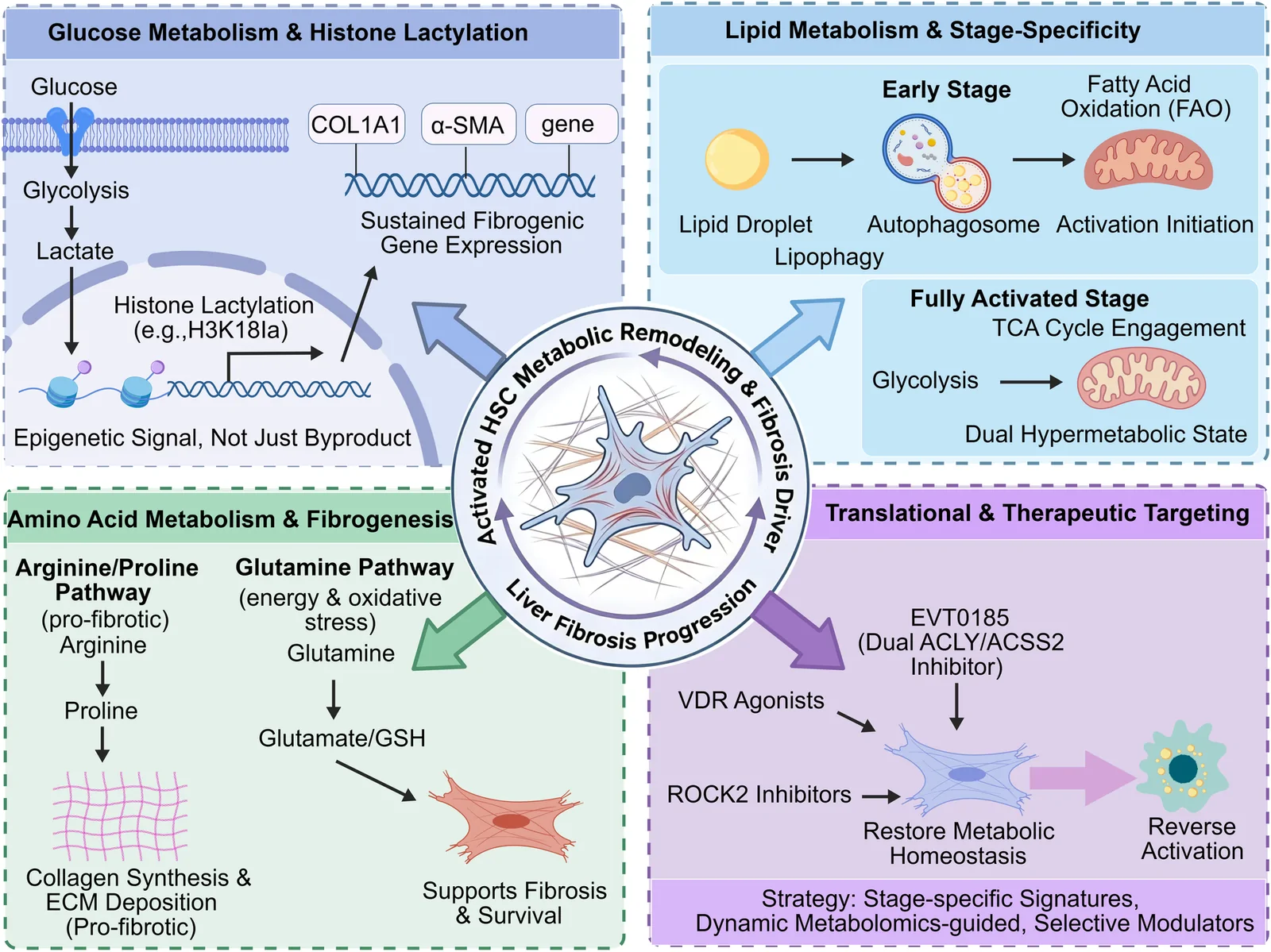
Metabolic reprogramming of HSCs and targeted therapeutic strategies in liver fibrosis.

**Figure 2.**
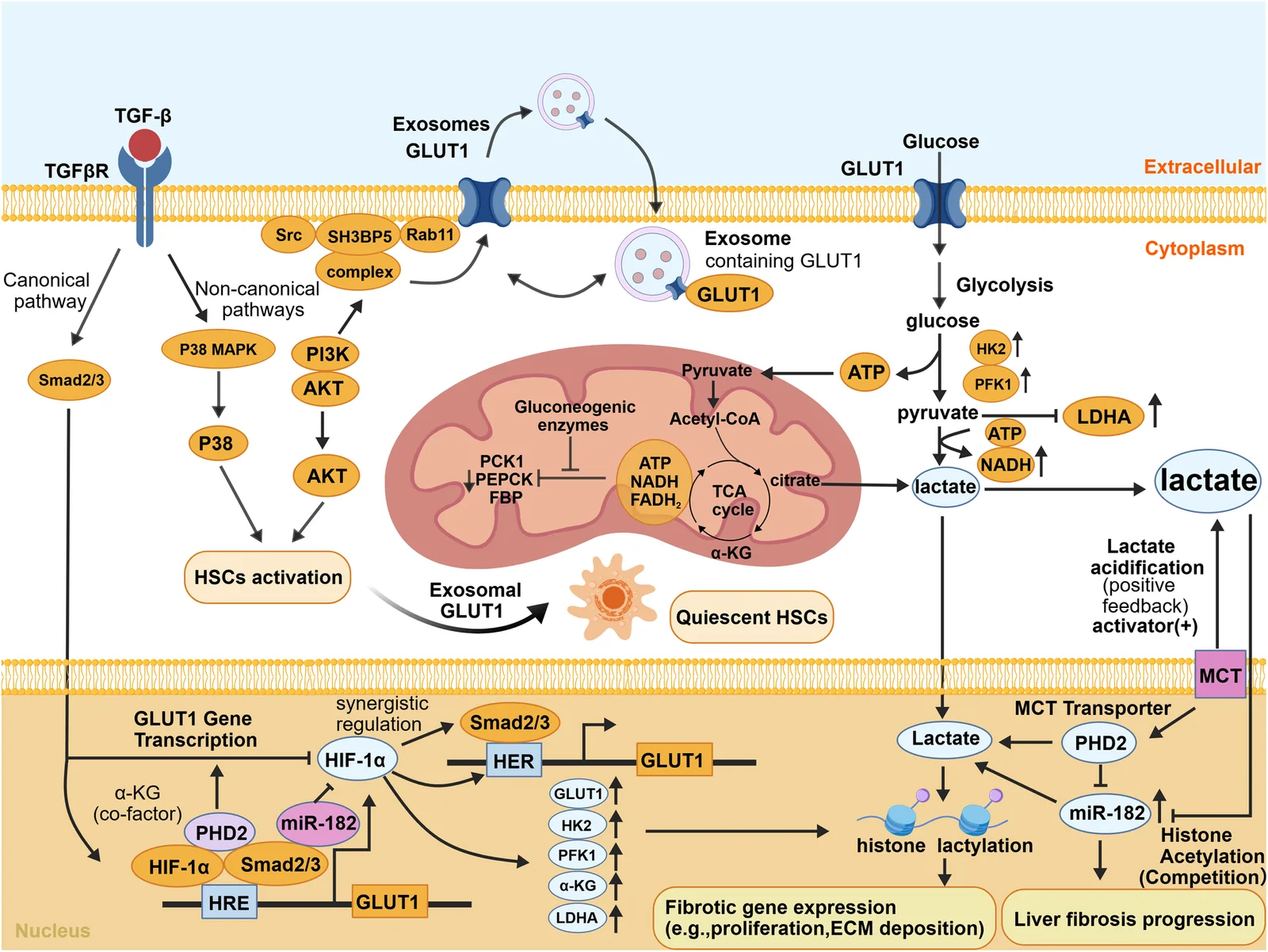
Signaling pathways involved in HSCs activation.

**Figure 3.**
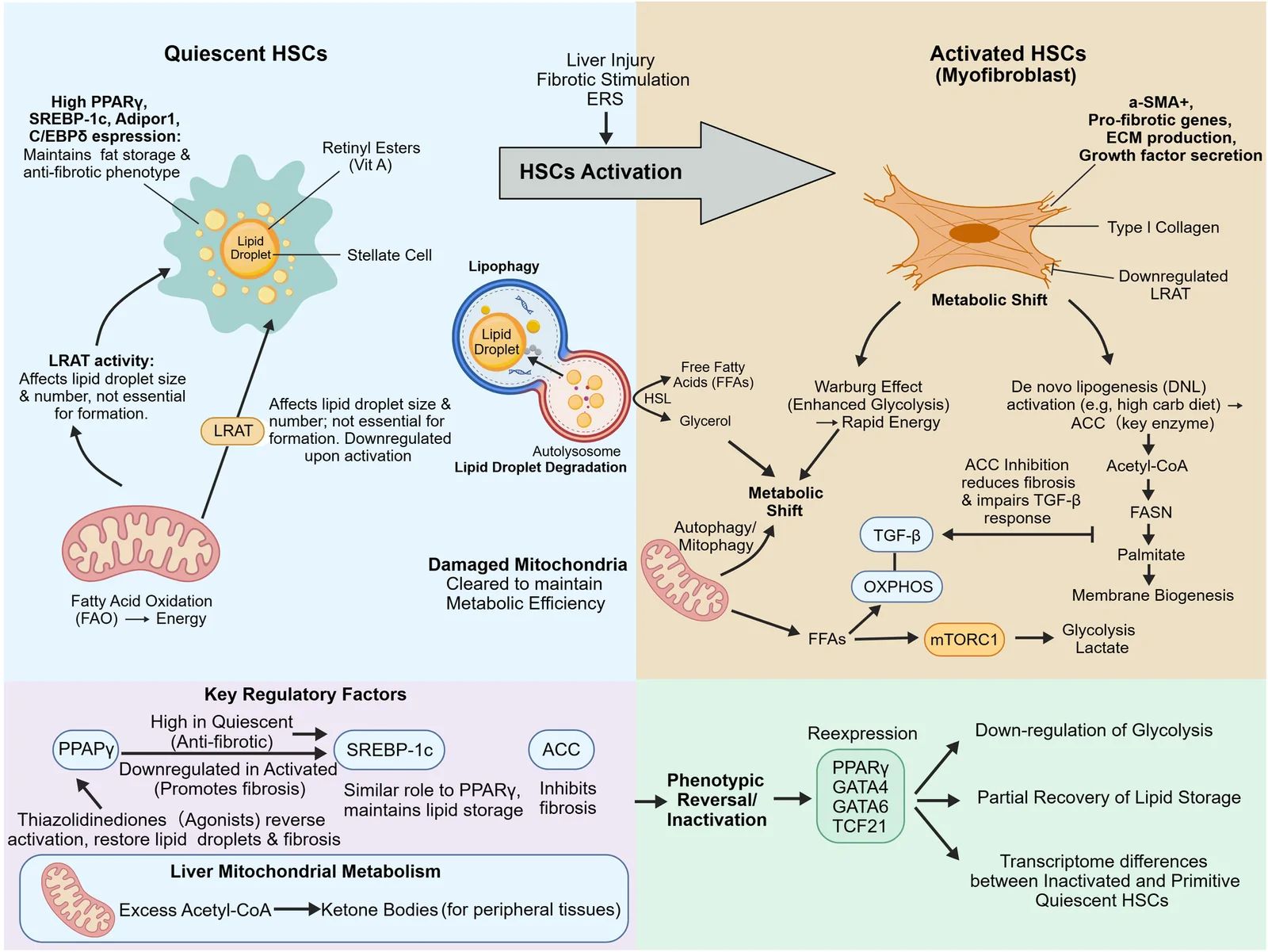
Glucose and lipid metabolic reprogramming in HSCs activation.

**Figure 4.**
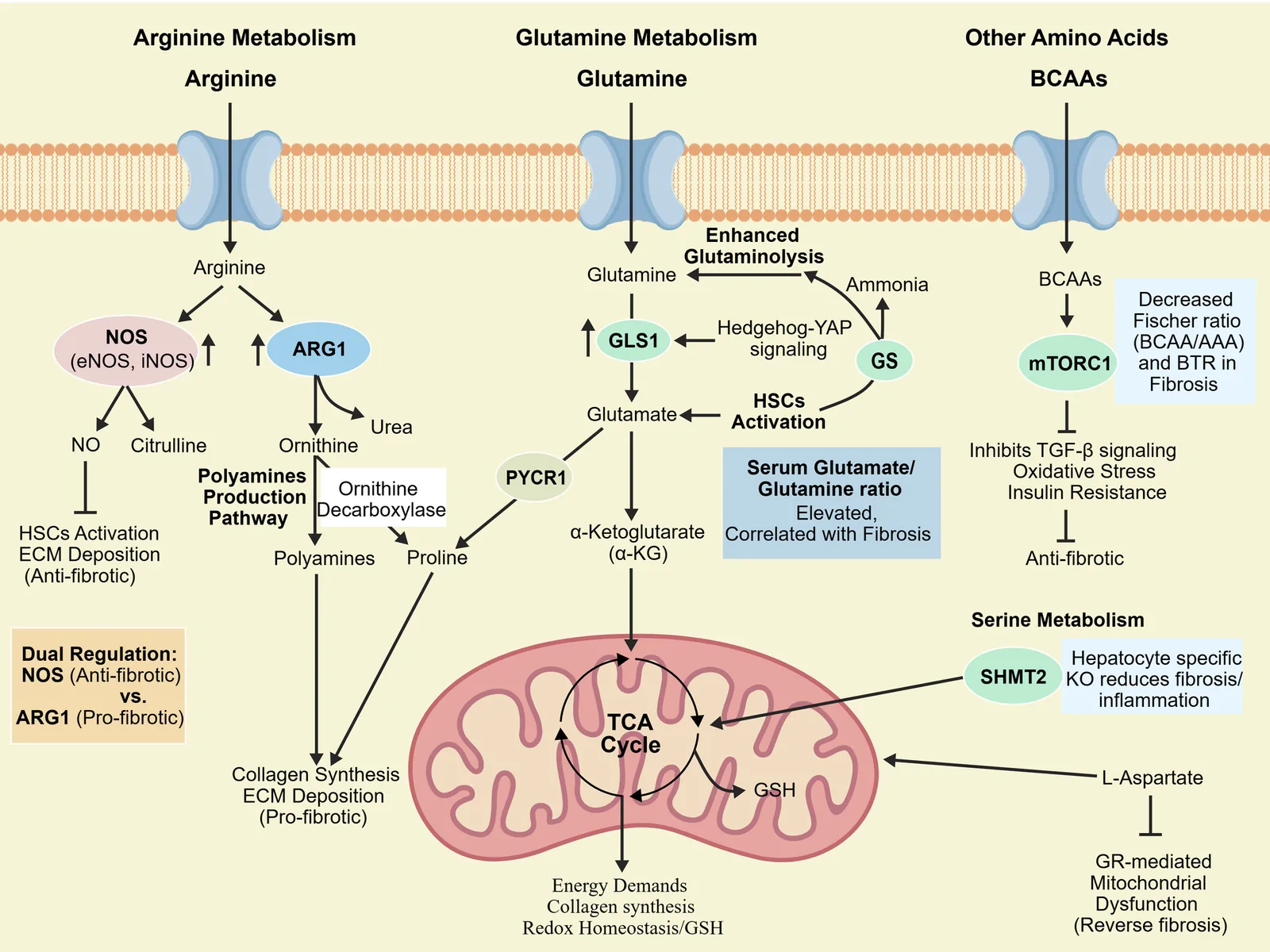
Changes in amino acid metabolism during HSCs activation.

Hepatic fibrosis is an inevitable stage in the development of cirrhosis, which is a severe consequence of fibrosis. Hepatic fibrosis is a core pathological response generated during the liver repair and healing process following various chronic injuries, such as viral hepatitis, alcoholic liver disease, and non-alcoholic steatohepatitis. However, persistent and excessive hepatic fibrosis ultimately leads to cirrhosis, characterized by diffuse liver fibrosis surrounding regenerative nodules. This leads to severe distortion of the normal lobular structure and vascular architecture (4). Progressive hepatic fibrosis is the common pathway through which nearly all chronic liver diseases lead to cirrhosis, which is the most important risk factor for the development of HCC (5). As the most common type of primary liver cancer, HCC continues to have high incidence and mortality rates worldwide. The persistent process of hepatic fibrosis, along with the associated remodeling of the extracellular matrix (ECM), creates a microenvironment that promotes inflammation, proliferation, and angiogenesis. This also provides favorable conditions for the malignant transformation of hepatocytes (6). Hepatic fibrosis is a pathological outcome of imbalanced repair following chronic liver injury. The core mechanism of liver fibrosis lies in the aberrant activation and metabolic reprogramming of hepatic stellate cells (HSCs). As the key driver cells of hepatic fibrosis, the phenotypic transition of HSCs from a quiescent to activated state is the central force driving fibrosis progression (7).

Metabolic reprogramming refers to the adaptive process by which tumor cells alter their metabolic pathways to meet energy demands and support biosynthesis under microenvironmental stresses such as hypoxia and nutrient deprivation. It has been recognized as one of the core hallmarks of cancer (8). Its typical phenotype is characterized by a preference for energy production through glycolysis even under oxygen-sufficient conditions (the Warburg effect), accompanied by a metabolic network reorganization that includes enhanced glutaminolysis and increased fatty acid synthesis (9). This metabolic shift not only provides ATP and biosynthetic precursors required for rapid tumor cell proliferation, but also remodels the tumor microenvironment through mechanisms such as lactate secretion, thereby promoting immunosuppression and therapy resistance (10). However, existing studies have shown that metabolic reprogramming is not limited to the field of oncology. It is also closely associated with various diseases, including liver fibrosis, kidney fibrosis, and pulmonary fibrosis (11–13). Therefore, in-depth investigation of the metabolic reprogramming of hepatic stellate cells during the development of liver fibrosis, the development of effective anti-fibrotic therapeutic strategies, and the blockade of its progression toward cirrhosis and HCC are of critical importance for improving the long-term prognosis of patients with chronic liver diseases.

This review systematically summarizes the research frontiers in the field of metabolic reprogramming of hepatic stellate cells. It focuses on the reprogramming processes that occur in glucose, lipid, and amino acid metabolism in HSCs, summarizes the mechanisms by which these metabolic alterations contribute to liver fibrosis progression, and discusses the clinical application prospects of targeting HSCs metabolism for the treatment of liver fibrosis.

Notably, while prior seminal reviews have outlined core metabolic features of activated HSCs (14, 15), this work distinguishes itself by three focused perspectives. First, it emphasizes stage-specific metabolic vulnerabilities across the quiescent, early-activated, fully activated, and reverting phases of HSCs, rather than treating activation as a uniform binary state. Second, it centers on the concept of metabolic homeostatic restoration as a therapeutic paradigm, shifting from non-specific metabolic suppression toward the targeted reversion of activated HSCs to a quiescent phenotype. Third, it integrates cutting-edge 2024–2026 evidence on gut microbiota-derived metabolites, cell-specific nanodelivery strategies, and early-phase clinical metabolic agents, providing an updated translational roadmap for anti-fibrotic drug development.

## 2 Literature search strategy

A narrative literature search was conducted across three electronic databases: PubMed, Web of Science, and Embase. The search covered publications from database inception to June 2026. Key search terms included combinations of “hepatic stellate cells”, “metabolic reprogramming”, “liver fibrosis”, “glycolysis”, “lipid metabolism”, “amino acid metabolism”, “histone lactylation”, “fibrosis reversal”, and “metabolic targeted therapy”. Inclusion criteria were: (1) peer-reviewed original research articles and review articles published in English; (2) studies focusing on metabolic reprogramming of HSCs in the context of liver fibrosis; (3) preclinical (*in vitro*, *in vivo*) and clinical studies investigating metabolic interventions for liver fibrosis. Exclusion criteria included conference abstracts, case reports, non-English publications, and studies without relevant mechanistic or translational data. The final reference list was curated based on relevance, methodological quality, and novelty, with priority given to recent high-impact studies published from 2020 to 2026.

## 3 Molecular mechanisms underlying the activation process of hepatic stellate cells

The activation of hepatic stellate cells (HSCs) is a core event in the progression of liver fibrosis. The traditional view describes it as a linear transformation from the resting state to a myofibroblast-like phenotype (16). However, recent studies suggest that this process is not linearly driven by a single signal pathway, but is determined by a complex interactive network composed of multiple signal pathways. Among them, the transforming growth factor -β (TGF-β)/Sma- and Mad-related protein (Smad) pathway dominates the transcriptional activation of matrix synthesis genes, while platelet-derived growth factor (PDGF) mainly regulates the proliferation and migration ability of HSCs through the p38 mitogen-activated protein kinase (MAPK) and phosphatidylinositol 3-kinase (PI3 K)/protein kinase B (AKT) signaling axes (17). It is worth noting that there is a functional synergy between these two pathways: The latest research shows that TGF-*β*1 concurrently activates the canonical Smad3 pathway and non-canonical PI3 K/Akt/mTOR pathway, and these two cascades form a positive feedback loop to mutually amplify HSCs activation (18). In addition to this pro-fibrotic network, the AMP-activated protein kinase (AMPK)-mitochondrial autophagy pathway has been confirmed in recent years to provide energy support for continuously activated HSCs (19). Studies have shown that the phosphorylation level of AMPK is upregulated during the activation of HSCs, maintaining mitochondrial autophagy and mitochondrial quality through the AMPK-unc-51 like autophagy activating kinase 1 (ULK1) and AMPK-regulatory-associated protein of mTOR (Raptor) pathways to ensure energy supply during the activation of HSCs (19). However, the role of AMPK in HSCs is context-dependent-studies have found that regulating AMPK in the pre-activation stage of HSCs can significantly affect the subsequent activation process, suggesting that AMPK plays a differentiated role in different stages of HSCs activation (19);. Meanwhile, retinoid X receptor *α* (RXR*α*) regulates HSCs activation by regulating AMPK*α* activation mediated by Ca2⁺/calmodulin-dependent protein kinase kinase *β* (CaMKK*β*), and this effect can be achieved by promoting mitochondrial autophagy (20). It is not clear whether this “metabolic adaptability” can be intervened.

From a critical perspective, there are still several key gaps in the current understanding of the activation mechanism of HSCs: First, most studies rely on *in vitro* culture-induced activation, ignoring the dynamic changes of cell-cell and cell-matrix interactions in the hepatic sinusoidal microenvironment; Recent single-cell transcriptomics studies have begun to reveal the heterogeneity and dynamic evolution trajectory of HSCs in different etiologies and durations of liver injury (21). However, how to translate these findings into precise intervention strategies remains a challenge. Secondly, there is still a lack of molecular-level evidence as to whether there are direct cross-regulatory nodes among different signaling pathways. Although some synergistic relationships among pathways have been identified, the specific molecular interaction mechanisms still require in-depth research (17). Thirdly, although existing studies have pointed out that the anti-fibrotic effect of single-pathway inhibitors is limited, the underlying reasons - whether it is pathway redundancy, compensatory activation, or insufficient selectivity of target cells - have not been systematically answered (17). Therefore, future research should shift from the “linear pathway superposition” model to the “dynamic network regulation” model, identify the key node molecules at different activation stages, and on this basis, design combined or sequential intervention strategies. Only in this way is it possible to achieve truly effective anti-fibrosis or fibrosis reversal.

## 4 Metabolic reprogramming during the activation process of hepatic stellate cells

### 4.1 Reprogramming of glucose transporters

Glucose transporter 1 (GLUT1), encoded by the SLC2A1 gene, is the main glucose transporter expressed in hepatic fibroblasts (22). In activated HSCs, the protein level of GLUT1 is significantly upregulated. This enhances glucose uptake across the cell membrane, leading to increased glycolysis. GLUT1 upregulation is a key upstream event in metabolic reprogramming. It is also an important therapeutic target for anti-fibrotic intervention - hypoxia inducible factor-1*α* (HIF-1*α*) is a core transcription factor in the hypoxic response. In the fibrotic liver microenvironment, it is stabilized and translocates into the nucleus, where it binds specifically to the hypoxia response elements (HREs) of target genes. HIF-1*α* significantly upregulates GLUT1 transcription by binding to its HRE. This enhances cellular glucose uptake and glycolysis (23). In the presence of HIF-1*α*, the expression of GLUT1 and key glycolytic enzymes, such as lactate dehydrogenase A (LDHA), hexokinase 2 (HK2), and phosphofructokinase-1 (PFK1), is significantly upregulated (24). Upon HIF-1*α* stimulation, activated HSCs secrete exosomes containing GLUT1. These exosomes are then taken up by non-activated HSCs, further promoting glucose uptake and glycolysis, thereby driving HSCs activation (25).

TGF-*β* is a key factor that promotes HSCs activation in liver fibrosis. It upregulates GLUT1 gene transcription through both the canonical Smad pathway and the non-canonical p38 MAPK/PI3 K/AKT pathways, thereby enhancing glucose transport:After TGF-*β* binds to its cell membrane receptor, it activates Smad2/3, which then forms a complex that translocates into the nucleus. This complex directly binds to the GLUT1 gene promoter to initiate its transcription. At the same time, TGF-*β* can also activate the p38 MAPK and PI3 K/AKT pathways. These two pathways operate independently of the Smad pathway. They enhance GLUT1 transcription efficiency by phosphorylating the downstream transcription factors (26). In addition, TGF-*β* stimulation affects the intracellular trafficking of GLUT1.Upon TGF-*β* stimulation, the Src SH3 domain binds to SH3BP5, forming a Src/SH3BP5/Rab11/GLUT1 complex. This complex promotes membrane trafficking and the localization of GLUT1, significantly enhancing glucose uptake and glycolysis in HSCs. Targeting the Src SH3 domain inhibits GLUT1 membrane localization and blocks HSCs activation (27).

### 4.2 Reprogramming of glucose metabolic pathways

Quiescent HSCs exhibit low glycolytic activity, and their glucose metabolism is primarily dependent on the oxidative phosphorylation pathway after HSCs activation, a metabolic shift similar to the Warburg effect in tumor cells occurs. Early activated HSCs prefer aerobic glycolysis and suppress oxidative phosphorylation even under oxygen-sufficient conditions. Although aerobic glycolysis produces ATP less efficiently than mitochondrial oxidative phosphorylation, this pathway can rapidly provide ATP and supply metabolic intermediates for biosynthesis (such as precursors for nucleotide and amino acid synthesis). This meets the synthetic demands of activated HSCs for proliferation and secretion of large amounts of extracellular matrix (14). Activated HSCs not only exhibit enhanced glycolysis, but also exhibit active or remodeled mitochondrial respiration (OCR) at certain stages to meet biosynthetic demands (15). Fully activated HSCs develop a unique “dual hypermetabolic” phenotype, in which glycolysis and tricarboxylic acid (TCA) cycle activity are simultaneously enhanced. Glucose-derived carbons are not only converted into lactate but also enter the TCA cycle in large amounts, leading to significantly elevated levels of citrate and *α*-ketoglutarate (*α*-KG). The downregulation of PCK1 rewires intracellular carbon metabolism, diverting metabolic intermediates toward the TCA cycle and supporting massive synthesis of collagen and lipids in activated HSCs. This metabolic remodeling eliminates the carbon flux toward gluconeogenesis and sustains the biosynthetic demands of HSCs (28). The shift in metabolic focus is also reflected in the following: as HSCs transition from the quiescent state to the activated aHSC state, the expression levels of key gluconeogenic enzymes, such as phosphoenolpyruvate carboxykinase (PEPCK) and fructose bisphosphatase (FBP), are significantly downregulated. This further confirms that metabolism shifts from the gluconeogenic mode to the glycolytic mode (29). In summary, both the glycolytic and TCA cycle pathways are enhanced during hepatic stellate cells activation, whereas the gluconeogenic pathway is reduced. This further indicates that HSCs shift from anabolic to catabolic metabolism to rapidly produce energy and accelerate their activation.

### 4.3 Reprogramming of glucose metabolites

During HSCs activation, glycolysis is hyperactive and the Warburg effect occurs, producing large amounts of lactate. Lactate is not only a hallmark end product of glucose metabolic reprogramming in HSCs but also an important metabolic signaling molecule that promotes sustained HSCs activation, generating a positive feedback effect (30). Recent studies have indicated that lactate plays a crucial role in the regulation of gene expression in HSCs. Lactate is not merely a metabolic byproduct, as traditionally thought, and is generated through HK2-mediated glycolysis. Lactate-mediated histone lactylation is one of the key epigenetic mechanisms maintaining fibrogenic gene expression in HSCs. Inhibiting HK2 or lactate production alleviated fibrosis, whereas lactate supplementation reversed this effect. Furthermore, histone lactylation and acetylation exhibit competitive regulatory effects. This reveals the key role of lactate in directly regulating epigenetic mechanisms through metabolic reprogramming (31). Lactate is exported out of the cell via monocarboxylate transporter (MCT) transporters, leading to the accumulation of extracellular lactate. This acidifies the cellular microenvironment, promotes HSCs activation, accelerates ECM deposition, and drives liver fibrosis progression (32). The acidic microenvironment resulting from lactate efflux further activates HIF-1*α* by inhibiting prolyl hydroxylase domain 2 (PHD2) (a negative regulator of HIF-1*α*) and enhancing the expression of microRNA-182 (miR-182) (a positive regulator of HIF-1*α*). In turn, activated HIF-1*α* induces the expression of more glycolytic enzymes and lactate transporters, ultimately reinforcing glycolysis, This positive feedback loop is also restricted by the negative regulation of monocarboxylate transporters (MCTs) (33). This enhanced glycolysis is not merely a concomitant phenomenon during HSCs activation but rather a key driving force and a necessary condition for initiating and maintaining the activated state.

Collectively, the findings from multiple studies indicate a close association between aberrant metabolic reprogramming of hepatic stellate cells and the progression of liver fibrosis. Among these, activated HSCs exhibit a hyperactivation of glucose metabolic pathways. Notably, this dramatic shift in energy metabolism is not only an important biomarker of HSCs transition to a pro-fibrotic phenotype but may also directly regulate the molecular mechanisms underlying their pathological activation. Therefore, by inhibiting the activity of glucose transporters, blocking the hyperactivity of the glycolytic pathway, or suppressing the production of the key metabolite lactate, we can slow down or even halt the activation of HSCs, thereby inhibiting the progression of liver fibrosis. This also represents a potential therapeutic direction for anti-fibrotic treatment.

## 5 Metabolic reprogramming of lipid metabolism

### 5.1 Lipid remodeling during the activation process of hepatic stellate cells

In a healthy liver, HSCs are in a quiescent state. The most notable feature of HSCs is that the cytoplasm is rich in retinol ester lipid droplets, which store 50% to 80% of the body's vitamin A. Quiescent HSCs express a group of characteristic lipid metabolism-related genes, including lecithin: retinol acyltransferase (LRAT), peroxisome proliferator-activated receptor *γ* (PPAR*γ*), adiponectin receptor 1 (Adipor1), and C/EBP*δ*, etc. Among them, LRAT is a key enzyme in retinol ester synthesis. The traditional view holds that it is crucial for lipid droplet formation in HSCs (34). However, the latest research indicates that LRAT mainly affects the size and quantity of lipid droplets rather than being absolutely necessary for lipid droplet formation (35). PPAR*γ*, as a member of the nuclear receptor superfamily, is highly expressed in resting HSCs and exerts anti-fibrotic effects by maintaining lipid storage and inhibiting the transcription of pro-fibrotic genes (36).

When the liver is stimulated by injury, HSCs rapidly enter the early activation stage. The most notable event at this stage is the rapid degradation of lipid droplets, which is mainly achieved through the selective autophagy pathway of lipophagy. Studies have confirmed that inducing autophagy can accelerate the loss of lipid droplets in quiescent HSCs and promote their activation, while blocking autophagy can inhibit the activation and fibrosis process of HSCs (37). The free fatty acids released by lipid droplet degradation enter the mitochondria for fatty acid oxidation (FAO), providing energy support for the early activation of HSCs (38).

After entering the continuous activation/proliferation stage, the metabolic pattern of HSCs undergoes a fundamental transformation. Activated HSCs exhibit a Warburg effect similar to that of tumor cells, that is, aerobic glycolysis is significantly enhanced, and they tend to generate energy through glycolysis rather than oxidative phosphorylation even under aerobic conditions. This metabolic conversion enables HSCs to rapidly produce ATP and biosynthetic precursors to meet their proliferation and synthesis of large amounts of extracellular matrix (ECM) requirements (14). Correspondingly, the *de novo* lipogenesis (DNL) pathway of fatty acids is upregulated, providing the lipid raw materials required for cell membrane construction. Acetyl-coa carboxylase (ACC), as the rate-limiting enzyme of DNL (39), has enhanced activity in activated HSCs. Studies have shown that inhibiting ACC not only reduces lipotoxicity but also impacts the ability of HSCs to respond to TGF-*β* -activated glycolysis, thereby inhibiting fibrosis formation by (40). However, with the complete activation of HSCs, the expression of LRAT was significantly down-regulated, and the cells then expressed pro-fibrotic genes such as *α* -smooth muscle actin (*α*-SMA) and type I collagen, presenting a typical myofibroblast phenotype (41, 42).

After liver injury stops, partially activated HSCs may undergo phenotypic reversal, that is, inactivation, and regain a quiescent phenotype. Some other activated HSCs are cleared through apoptosis or senescence pathways (43). Studies have shown that the reexpression of transcription factors PPAR*γ*, GATA4, GATA6 and TCF21 can promote the reversal of HSCs to a quiescence state (38). This process is also accompanied by the reversal of metabolic patterns, including the down-regulation of glycolysis and the partial recovery of lipid storage capacity, but there are still transcriptome differences between inactivated HSCs and primitive quiescent HSCs (43). This discovery provides an important theoretical basis for the development of therapeutic strategies that promote the reversal of fibrosis.

### 5.2 Alterations of key regulatory factors during the reprogramming process of lipid metabolism

Peroxisome proliferator-activated receptors (PPARs) play an indispensable role in maintaining liver homeostasis, regulating fatty acid oxidation, triglyceride metabolism and adipogenesis. On the one hand, PPARs can keep HSCs in a quiescent state and regulate inflammatory responses, thereby effectively preventing the occurrence of liver fibrosis. On the other hand, PPARs can enhance insulin sensitivity while regulating the storage of triglycerides in adipose tissue (36). Peroxisome proliferator-activated receptor *γ* (PPAR-*γ*) is a member of the nuclear hormone receptor superfamily. As a transcription factor that can be activated by ligands, it plays a key role in liver lipid metabolism and inflammatory responses, and is an important factor for liver metabolic regulation (44). It is also a core lipid sensor involved in lipid homeostasis and cell fate regulation (45). It is highly expressed in quiescent HSCs and inhibits activation. Its down-regulation promotes lipid droplet loss and fibrosis (46). PPAR*γ* agonists (such as thiazolidinediones) can restore lipid droplet storage, reverse HSCs activation and liver fibrosis (47).

### 5.3 Alterations in metabolic pathways during lipid metabolism reprogramming

The metabolic reprogramming of HSCs is also related to autophagy: autophagy is a process by which cells break down their own components to provide energy and raw materials, which is important for maintaining cellular homeostasis (48). Autophagy directly affects the activation status and fibrosis process of HSCs by regulating energy metabolism, lipid dynamics and signaling pathways (49). In HSCs, autophagy may be involved in its activation process, especially in the decomposition of lipid droplets. Autophagy tilts towards glucose metabolism. The fatty acids released by autophagy in the decomposition of lipid droplets can inhibit glucose oxidation and promote glycolysis (Warburg effect). The enhancement of glycolysis further depends on autophagy to clear damaged mitochondria (mitochondrial autophagy) and maintain metabolic efficiency. Provide rapid energy for activated HSCs (50). Endoplasmic reticulum stress (ERS) can activate autophagy, thereby promoting the decomposition process of lipid droplets and facilitating the release of fatty acids. The released fatty acids not only can inhibit oxidative phosphorylation, but also can increase glycolytic flux by activating the mammalian target of rapamycin complex 1 (mTORC1) signaling pathway, thereby ensuring that HSCs remain in an activated state continuously (51).

In conclusion, during the process of liver fibrosis, the activation of hepatic stellate cells is accompanied by a significant remodeling of lipid metabolism. These cells undergo lipid droplet degradation and metabolic mode conversion, enhance fatty acid oxidation energy supply through lipophagy, and then shift to the Warburg effect dominated by glycolysis and upregulate fatty acid synthesis, providing energy and membrane construction raw materials for the fibrosis process. Meanwhile, the high expression of PPAR*γ* and SREBP-1C in the quiescent state is crucial for maintaining lipid storage and anti-fibrotic status, while the down-regulation of their expression during activation drives lipid droplet loss and the transformation of pro-fibrotic phenotypes. This suggests that the core role of lipid metabolism pathways in the activation of hepatic stellate cells cannot be ignored.

## 6 Metabolic reprogramming of amino acid metabolism

### 6.1 Arginine metabolism

During the process of liver fibrosis, amino acid metabolism is reprogrammed in a cell-type-specific manner, with effects on HSCs mediated by both HSC-intrinsic metabolic rewiring and indirect paracrine or systemic metabolic alterations. The following subsections detail these pathways, with explicit distinction between cell-autonomous and non-autonomous mechanisms. Arginine metabolism mainly occurs through two competitive pathways: on the one hand, nitric oxide synthase (NOS) catalyzes arginine to generate nitric oxide (NO) and citrulline; on the other hand, arginase decomposes arginine into ornithine and urea (52).

[HSC-intrinsic mechanism] Within HSCs, NO produced by endothelial NOS (eNOS) in resting HSCs maintains the quiescent state of cells (53); In activated HSCs, the expression of inducible NOS (iNOS) is downregulated, while arginase 1 (ARG1) is significantly upregulated, leading to the flow of arginine into ornithine and polyamine production pathways, promoting collagen synthesis and ECM deposition (54). In addition, arginine can be converted into ornithine through the urea cycle and further transformed into proline (55),. which is the main component of collagen and directly promotes the fibrosis process (56). The regulatory effect of arginine metabolites on liver fibrosis shows a significant duality, which can be explained by the “metabolite balance hypothesis”: when arginine metabolism flows to the NO synthesis pathway (dominated by NOS), it mainly plays an inhibitory role in fibrosis. At this time, NO can exert an anti-fibrotic effect by inhibiting the activation of HSCs and the deposition of ECM. When it flows towards the ornithine - polyamine/proline metabolic axis (dominated by ARG1), it tends to promote the fibrosis process. Polyamine and proline, as downstream products of ornithine metabolism, their pro-fibrotic effect is mainly reflected in their role as key precursors for collagen synthesis, directly promoting the accumulation of ECM and thereby exacerbating liver fibrosis.

[Indirect immune-mediated mechanism] It is worth noting that this dual regulation is also regulated by immune cells: when macrophages express ARG1, they can indirectly alleviate liver fibrosis by consuming arginine to inhibit the production of Th2-type cytokines and T cell proliferation. Conversely, when macrophage-specific ARG1 is absent, the level of Th2-type cytokines significantly increases, leading to an aggravation of liver fibrosis (57). The action directions of these two types of cells are different: the upregulation of ARG1 endogenous in HSCs directly promotes collagen synthesis, while ARG1 derived from macrophages indirectly exerts anti-fibrotic effects through immune regulation. Moreover, after the activation of HSCs, the ARG1 activity is higher than that in the resting state. This change promotes the catabolism of arginine, which in turn generates key metabolites such as polyamines and proline (54).

### 6.2 Glutamine metabolism

[HSC-intrinsic mechanism] Glutamine catabolism (catalyzed by glutaminase GLS to generate glutamic acid) plays a core role in the activation of HSCs and is one of the most thoroughly studied amino acids in liver fibrosis. Within the HSCs, its metabolic reprogramming is mainly manifested as enhanced glutamine catabolism (mediated by glutaminase) and mitochondrial metabolic dependence. Du et al. found in a non-alcoholic steatohepatitis (NASH) model that the expression of glutaminase 1 (GLS1) was significantly upregulated in fibrotic livers and was mainly located in activated HSCs, leading to an increase in the serum glutamate/glutamine ratio. This ratio was positively correlated with the degree of liver fibrosis and the content of myofibroblasts (58). Mechanistically, the Hedgehog-YAP signaling pathway activates GLS1 through transcription, promoting the decomposition of glutamine into *α* -ketoglutaric acid (*α*-KG), providing *α*-KG for the TCA cycle, maintaining the energy requirements and collagen synthesis of HSCs, and simultaneously maintaining the intracellular level of reduced glutathione to resist oxidative stress (59). In the biliary ligation (BDL) model, inhibition of GLS1 can reduce the expression of *α*-SMA and Col1a1 and alleviate liver fibrosis (60).

In addition to the intrinsic mechanism of HSCs, the disorder of the glutamine-ammonia-urea cycle axis leads to ammonia accumulation and an increase in ammonia concentration in the liver. This change stimulates glutamine synthase (GS) to promote the synthesis of glutamine, and the elevated glutamine can further enter the cell through the transport protein on the HSCs cell membrane, activating HSCs (61). This part involves the indirect regulation of HSCs by systemic ammonia metabolism.

### 6.3 Metabolism of other amino acids

[HSC-intrinsic mechanism] Proline is a key precursor for collagen synthesis, and its metabolism is significantly upregulated during fibrosis. In activated HSCs, glutamine-derived glutamic acid is converted into proline through pyrroline-5-carboxylic acid reductase (PYCR1), providing raw materials for ECM. Studies have shown that inhibiting PYCR1 can reduce collagen deposition in liver fibrosis models (62).

[Indirect systemic/paracrine mechanism] Branched-chain amino acids (BCAAs) are not only nutritional substrates for protein synthesis, but also play a significant role in nutritional metabolism, energy homeostasis and immune response through multiple signaling pathways. From the perspective of the interaction between systemic metabolism and HSCs, BCAAs have been proven to improve liver fibrosis by inhibiting TGF-*β* signaling in HSCs in an mTORC1-dependent manner, reducing oxidative stress and improving insulin resistance (63). In clinical studies, the Fischer ratio (branched-chain amino acids/aromatic amino acids) of patients with liver fibrosis decreased, and the ratio of branched-chain amino acids to tyrosine (BTR) significantly decreased with the progression of fibrosis (64). This reflects systemic changes in the amino acid profile rather than intrinsic metabolic alterations in HSCs.

[Indirect hepatocyte-mediated mechanism] In addition to the above-mentioned amino acids, the serine metabolic pathway is also involved in liver fibrosis. However, it should be noted that Chen et al. found that specific knockout of serine hydroxymethyltransferase 2 (SHMT2) in hepatocytes can reduce liver inflammation and fibrosis (65). This effect mainly stems from the indirect regulation of HSCs by the metabolic remodeling of hepatocytes. Rather than the inherent SHMT2 function of HSCs.

[Indirect systemic/hepatocyte-mediated mechanism] In addition, L-aspartic acid-related metabolism may also be involved. Studies have found that L-aspartic acid can reverse carbon tetrachloride-induced liver fibrosis by inhibiting glucocorticoid receptor (GR)-mediated mitochondrial dysfunction (66), suggesting that this non-essential amino acid may indirectly interfere with the fibrosis process by affecting mitochondrial function throughout the body or in liver cells.

In summary, during the process of liver fibrosis, the activation of HSCs is accompanied by significant amino acid metabolic reprogramming. Together with lipid metabolic remodeling (lipid droplet degradation, fatty acid oxidation to glycolysis and lipid synthesis conversion) and hyperactivity of glucose metabolic pathways, they constitute the core metabolic characteristics of the phenotypic transformation of HSCs promoting fibrosis. Specifically: The arginine metabolic axis is bidirectionally regulated within HSCs (NOS vs. ARG1), and is simultaneously immunologically regulated by macrophage ARG1. Glutamine catabolism is significantly enhanced within HSCs, providing support for the TCA cycle and antioxidation. Proline synthesis is crucially upregulated in activated HSCs; Metabolic pathways such as branched-chain amino acids, serine (mainly through liver cell SHMT2), and aspartic acid mainly indirectly regulate HSCs function through systemic metabolism or paracrine mechanisms. These various amino acid metabolic reconfigurations, like hyperglycolytic activation and lipid metabolism conversion, are not only key indicators of the activation status of HSCs, but also the intrinsic molecular mechanisms that directly drive their pro-fibrotic functions such as collagen synthesis, proliferation and survival. Therefore, targeted regulation of the intrinsic key amino acid metabolic enzymes (such as GLS1, ARG1, and PYCR1) in HSCs, or indirect influence on the function of HSCs by intervening in the metabolism of hepatocytes/macrophages, can effectively intervene in the process of liver fibrosis.

## 7 Metabolic reprogramming governing the reversion of activated hepatic stellate cells to quiescence

### 7.1 Reversibility of HSCs activation: a new perspective

As detailed in Sections 2–4, HSCs activation is driven by profound metabolic remodeling. Early studies suggested that this activation is irreversible (67), but accumulating evidence demonstrates that upon injury resolution or metabolic intervention, activated HSCs can return to a quiescent-like state (68). Phenotypic reversion is now recognized as a core mechanism of fibrosis regression. Importantly, reversion is not simply the opposite of activation; it involves distinct molecular events that do not fully recapitulate the original quiescent state. This section focuses on the reversion-specific metabolic processes and their therapeutic implications.

### 7.2 Glycolysis suppression and oxidative phosphorylation recovery

While activation enhances glycolysis (Section 2.2), reversion requires suppression of glycolytic flux and restoration of oxidative phosphorylation(OXPHOS). Inhibiting key glycolytic enzymes such as PKM2 or HK2 in activated HSCs reduces lactate production and histone lactylation (H3K18la), thereby silencing fibrogenic genes like ACTA2 and COL1A1 (69). Unlike activation, where the TCA cycle is also engaged (Section 2.2), the reversion process depends on a functional switch from aerobic glycolysis back to mitochondrial OXPHOS, although the exact metabolic signature of fully reverted HSCs remains incompletely defined (15).

### 7.3 Lipid droplet re-accumulation and the PPAR*γ*/LRAT axis

Activation causes lipid droplet degradation via lipophagy (Section 3.1). In reversion, the key event is PPAR*γ*-dependent lipid droplet regeneration. PPAR*γ* expression is upregulated, which activates lipogenic genes (SREBP-1c) and restores LRAT activity, leading to re-esterification of vitamin A and re-accumulation of retinyl ester droplets (15) HSCs-specific PPAR*γ* knockout impairs fibrosis resolution *in vivo* (70), underscoring that restoring PPAR*γ* activity is a non-mirroring, therapeutically actionable step that is not merely the reverse of activation (where PPAR*γ* is suppressed).

### 7.4 Normalization of glutamine metabolism and epigenetic memory erasure

Activated HSCs exhibit GLS1-driven glutaminolysis (Section 4.2). In reversion, GLS1 downregulation reduces *α*-KG production, alleviating epigenetic pressure (71). A unique feature of HSCs reversion is the erasure of metabolic-epigenetic memory, including the removal of histone lactylation (e.g., H3K18la) and DNA methylation modifications that sustain the fibrotic phenotype. While reduced lactate production represents a passive reversal of the activated metabolic state, the active enzymatic mechanisms underlying histone delactylation during HSCs reversion remain largely unexplored. In other cellular contexts, histone deacetylases (HDACs) and sirtuins (SIRTs) have been identified as potential histone delactylases, yet their specific roles and regulatory mechanisms in HSCs deactivation are unknown and warrant future investigation (31, 72).

### 7.5 Interplay between metabolism and signaling pathways during reversion

During the progression of liver fibrosis, activated HSCs undergo profound metabolic reprogramming characterized by enhanced glycolysis and glutaminolysis (15, 71, 73). The resulting accumulation of lactate and *α*-ketoglutarate (*α*-KG) not only fulfills biosynthetic demands but also amplifies TGF-*β*/Smad and PI3 K/Akt signaling cascades through epigenetic mechanisms such as histone lactylation and DNA methylation, thereby stabilizing the myofibroblastic phenotype (15, 31). Conversely, during the reversion of HSCs toward a quiescent-like state, restored oxidative phosphorylation and lipid droplet accumulation cooperate to suppress TGF-*β*/Smad signaling, while transcription factors such as PPAR*γ*, GATA6, and TCF21 promote and maintain the deactivated phenotype (15, 70). Notably, HSCs-specific deletion of PPAR*γ* significantly impedes fibrosis resolution *in vivo*, underscoring the critical role of metabolic normalization in HSCs deactivation (70). The bidirectional crosstalk between metabolic reprogramming and classical profibrotic pathways thus creates a dynamic regulatory network that determines HSCs fate (15). Targeting specific metabolic nodes within these signaling axes—such as glycolytic enzymes, glutaminase 1, or PPAR*γ* agonists — has emerged as a promising anti-fibrotic strategy to induce HSCs reversion and promote the resolution of liver fibrosis (15, 31, 71, 73).

### 7.6 Regulation of HSC activation reversibility by gut microbial metabolites

When discussing the reversibility and plasticity of hepatic stellate cell (HSCs) activation, it is worthwhile to incorporate the emerging perspective of gut microbiota-derived metabolites regulating HSCs phenotypic switching via the gut-liver axis. The transdifferentiation of quiescent HSCs into activated myofibroblasts is a central event that initiates liver fibrosis, whereas whether activated HSCs can revert to a quiescent-like phenotype enriched with lipid droplets is a key scientific question for achieving fibrosis resolution. Of note, to date no gut microbial metabolite has been proven to stably mediate the complete return of fully activated HSCs to their original quiescent state; however, multiple metabolites can inhibit sustained HSCs activation and promote the transition of activated HSCs toward a deactivated phenotype with reduced fibrogenic activity.

Representative regulatory molecules include short-chain fatty acids (SCFAs), secondary bile acids, and tryptophan-derived metabolites such as indole-3-propionic acid (IPA). Among SCFAs, butyrate has the most robust evidence: it blocks non-canonical TGF-*β* signaling via histone deacetylase (HDAC) inhibition, suppresses aerobic glycolysis reprogramming in HSCs, and downregulates *α*-SMA and type I collagen expression. *In vitro* models demonstrate its potential to drive activated HSCs toward a deactivated phenotype and promote fibrosis regression, and in NASH-associated liver fibrosis animal models it significantly reduces collagen deposition (74). Propionate also exhibits anti-fibrotic effects, but its action is mainly to block *de novo* HSCs activation, with relatively limited evidence for directly inducing phenotypic reversal of activated HSCs (75). Secondary bile acids display a clear bidirectional regulatory profile: FXR/TGR5 agonistic bile acids can inhibit the pro-fibrotic program in HSCs, whereas subtypes such as deoxycholic acid can exacerbate HSCs activation. This suggests that the overall bile acid profile rather than any single molecule determines the net effect, and they are unlikely to independently drive HSCs phenotypic reversal (76). The regulation of HSCs plasticity by the tryptophan-derived metabolite IPA is notably controversial. *In vitro* studies show that IPA at appropriate concentrations can remodel mitochondrial metabolism in activated HSCs, inhibit migration and fibrogenic gene transcription, and directly drive activated HSCs into an inactive state (77). However, other experiments have found that under specific culture conditions, IPA promotes HSCs activation via the ROS/JNK/p38 pathway (78). Further *in vivo* mechanistic studies suggest that IPA alleviates liver fibrosis more indirectly by targeting pro-fibrogenic hepatic macrophages and attenuating the macrophage-HSCs pro-fibrotic paracrine loop, rather than by directly inducing HSCs phenotypic reversal (79). These discrepancies indicate that IPA's function is highly dependent on concentration, microenvironment, and injury model.

Overall, gut microbial metabolites constitute a distal gut-liver regulatory network involved in modulating the activation-deactivation homeostasis of HSCs. However, most of these molecules only block sustained activation or promote partial phenotypic regression; they are insufficient to completely erase the epigenetic memory of activated HSCs or achieve full quiescent phenotypic reversal. Incorporating gut-liver axis microbial metabolic signals into the framework of HSCs plasticity can broaden the traditional research perspective that focuses mainly on local hepatic cytokines, providing new insights into elucidating the barriers to activated HSCs phenotypic reversal and developing microbiota-targeted adjuvant strategies for liver fibrosis.

## 8 Clinical application prospects of targeting hepatic stellate cell metabolism

The concept of metabolic reprogramming of HSCs is equally important in clinical practice, providing a new therapeutic entry point for clinical treatment. The current research focus has shifted from merely inhibiting matrix synthesis to promoting the reversal of activated HSCs to the resting phenotype and simultaneously enhancing the degradation of extracellular matrix. Intervention strategies targeting HSCs metabolism are gradually emerging as new approaches to reverse liver fibrosis, especially for patients with metabolism-related fatty liver disease (MASLD/MASH) and early liver cirrhosis. Currently, multiple drugs have entered the clinical trial stage (38, 80). There are significant differences among various drugs in terms of their mechanism of action, clinical development stage, and direct correlation with HSCs. They can be classified into three categories: systemic MASH-associated metabolic therapy, indirect anti-fibrotic therapy, and more direct HSCs metabolic targeted therapy.

### 8.1 Systemic metabolic therapies for MASH (indirect HSC modulation)

Agents in this category primarily act by improving systemic metabolic homeostasis, hepatic steatosis, and insulin sensitivity. They do not directly target the intrinsic glucose, lipid, or amino acid metabolic circuits within HSCs, and their anti-fibrotic effects are largely secondary to reduced hepatic injury and inflammation. Resmetirom is the first drug approved for the treatment of metabolic dysfunction-associated steatohepatitis (MASH) and related fibrosis. Resmetirom is a thyroid hormone receptor *β* agonist (THR-*β*) that can improve at least one stage of liver fibrosis in adult patients with non-cirrhotic NASH or those with moderate to severe liver fibrosis, but most patients experience mild adverse reactions (81). This agent primarily targets hepatic lipid-metabolism pathways and exerts indirect effects; it lacks direct modulatory activity on intrinsic HSCs glucose or amino-acid metabolism. Tirzepatide is a glucagon-like peptide-1 receptor (GLP-1R)/glucose-dependent insulinotropic polypeptide receptor (GIPR) dual receptor agonist that can effectively improve the degree of liver fibrosis in MASH patients with F2 or F3 (moderate or severe) fibrosis, but it is often accompanied by gastrointestinal adverse reactions. Pegozafermin is an fibroblast growth factor 21 (FGF21) analogue similar to Tirzepatide, which improves fibrosis while accompanied by gastrointestinal side effects (82). Survodutide exerts its effect through the dual activation of glucagon receptor and GLP-1 receptor, and while improving liver fibrosis, it also has adverse gastrointestinal reactions (83). These systemic metabolic agents mainly remodel whole-body lipid homeostasis and insulin sensitivity, without directly targeting HSC-intrinsic glucose or amino-acid metabolic circuits. Most of these traditional standard treatment drugs can only improve liver fibrosis but are difficult to reverse or prevent its development. Moreover, most of them are still in the clinical trial stage, lacking a large number of clinical samples for verification. At the same time, they are accompanied by side effects and lack direct intervention ability for activated HSCs and their series of combined reactions.

### 8.2 Indirect anti-fibrotic therapy

Rifaximin-α slows down the progression of fibrosis by repairing the intestinal barrier and reducing inflammation, but it does not promote the reversal of liver fibrosis (84). Studies have shown that rifaximin combined with lubiprostone can significantly inhibit macrophage expansion, pro-inflammatory response and liver fibrosis by blocking the liver translocation of lipopolysaccharide and the activation of Toll-like receptor 4 signaling pathway (85).

### 8.3 Direct anti-fibrotic therapy targeting HSC-lntrinsic metabolism

Agents in this category directly modulate key metabolic nodes within HSCs (e.g., glycolytic enzymes, lipid synthesis pathways, epigenetic metabolic signals) to drive HSCs deactivation or quiescence restoration. Targeting the metabolism of HSCs for the clinical treatment of liver fibrosis is a new strategy, that is, regulating the energy metabolism of HSCs (such as glycolysis) fundamentally induces activated cells to return to a resting state. Recent studies have alleviated liver fibrosis by targeting key metabolic pathways mediated by nanomaterials: Li's research developed paeoniflorin-loaded copper-doped ZIF-8 nanomaterials targeting activated HSCs. This candidate agent targets HSCs glucose metabolism: This material inhibits the activity of key glycolytic enzymes by releasing Cu2⁺/Zn2⁺, blocks the energy metabolism of activated HSCs, and suppresses the secretion of their exosomes, thereby blocking the side-activation effect of activated HSCs on resting HSCs. This strategy significantly alleviated liver fibrosis in the preclinical model (86). The recently described dual ACLY/ACSS2 inhibitor EVT0185, which targets acetyl-CoA homeostasis in lipid metabolism, has demonstrated potent anti-fibrotic activity in human primary HSCs and preclinical murine models of MASH. It is currently in Phase 1 clinical evaluation and represents one of the most advanced metabolic-targeted agents in development for liver fibrosis (87). Vitamin D receptor (VDR), as a key transcriptional coordinator in fatty acid metabolism, its activation can down-regulate fat synthases such as FASN and ACC1, and up-regulate CPT (fatty acid oxidation rate-limiting enzyme). The natural VDR agonist extracted from Ligusticum chuanxiong targets HSCs lipid metabolism reprograms the fatty acid metabolism of HSCs through dual regulation (inhibiting synthesis and promoting oxidation). The down-regulation of VDR under pathological conditions is a key link in the activation of HSCs, and the activation of VDR can reverse the abnormal phosphorylation and metabolism of Smad3 induced by TGF-*β*, which provides a theoretical basis for the development of VDR agonist anti-fibrotic drugs (88). Fibroblast growth factor 21 (FGF21) is a key hormone regulating energy metabolism and has a direct anti-fibrotic effect in liver fibrosis. FGF21 analogues exert their effects through multiple targets - directly inhibiting HSCs activation, improving insulin sensitivity, promoting fatty acid oxidation, and alleviating endoplasmic reticulum stress. Clinical trials have shown that Efimosfermin alfa (BOS-580) is an FGF21 analogue acting predominantly on lipid-metabolism signalling. After 24 weeks of treatment, the degree of liver fibrosis was relieved and the overall tolerance was good (89). diacylglycerol O-acyltransferase 2 (DGAT2) is the rate-limiting enzyme that catalyzes the final step of triglyceride synthesis. In preclinical models, the use of antisense oligonucleotide ION224 to inhibit DGAT2 targets lipid-metabolism pathways can suppress the formation of new fat, reduce fat accumulation in the liver, improve liver steatosis, block the activation of HSCs caused by lipotoxicity, alleviate the progression of liver fibrosis, and there are no serious adverse events related to treatment in clinical practice, nor metabolic side effects such as hypertriglyceridemia. This drug blocks the activation of HSCs by targeting lipid metabolism (90). TDI01 is a highly selective Rho-associated coiled-coil kinase 2 (ROCK2) inhibitor that targets the perivascular hepatic microenvironment; it is currently in Phase 2 clinical trials for liver fibrosis, representing the first agent in this mechanistic class to enter clinical development. Notably, TDI01 does not directly act on intrinsic glucose, lipid, or amino acid metabolic enzymes in HSCs; it suppresses HSCs activation indirectly by remodeling the perivascular hepatic microenvironment (91).

The above-mentioned metabolic targeting strategy marks that the treatment of liver fibrosis is shifting from “passive defense” to “active intervention”. However, current research overly focuses on inhibiting the abnormal metabolism of activated HSCs, while neglecting the fundamental issue of how resting HSCs maintain metabolic homeostasis in the long term. The reversal of activated HSCs to the resting state is not simply the closure of the glycolytic pathway, but rather requires the restoration of a delicate balance between mitochondrial fatty acid oxidation and low-level glycolysis. Therefore, future drug design should shift from a strategy of “metabolism-targeted inhibition” to “metabolic homeostasis restoration”, providing metabolic support signals for HSCs to restore their resting phenotype. Meanwhile, dynamic metabolomics and single-cell metabolic flux analysis should be introduced into the endpoints of traditional clinical trials to assess the overall repositioning of HSCs metabolism, rather than merely observing the improvement in the fibrosis stage. Only by achieving a new transformation from “metabolic targeting” to “metabolic homeostasis” can we prevent “metabolic intervention” from turning into a new “mechanism-based killing” and truly achieve functional cure.

## 9 Future prospects

Liver fibrosis is a complex pathological process that is co-regulated by multiple cell types and signaling pathways. The metabolic reprogramming of hepatic stellate cells is a core regulatory link in this process. Upon liver injury, quiescent HSCs undergo a phenotypic switch to an activated state, accompanied by multiple metabolic reconfigurations involving glucose, lipids, and amino acids. These changes not only provide key energy and biosynthetic precursors for HSCs proliferation and extracellular matrix synthesis and secretion, but also amplify the activation effects of fibrotic signaling pathways through metabolite-mediated epigenetic regulation and intercellular communication. An in-depth exploration of the mechanisms underlying HSCs metabolic reprogramming is a key entry point for alleviating the occurrence and progression of liver fibrosis.

Targeting metabolic reprogramming to restore quiescence — a new paradigm for fibrosis reversal. A critical emerging frontier is to therapeutically restore the quiescent metabolic state in activated HSCs rather than only inhibiting their hypermetabolic phenotype. Future anti-fibrotic strategies should prioritize inducing metabolic reversion by rebuilding lipid droplets, restoring PPAR*γ* activity, and suppressing glycolysis and lactate-driven epigenetic modifications, as well as developing “reversal-selective” modulators that direct activated HSCs back toward quiescence instead of inducing cell death. Meanwhile, defining the specific metabolic signature of reverted HSCs using single-cell metabolomics will help identify stage-specific intervention targets, and combining metabolic modulation with pro-resolution signals will simultaneously promote HSCs inactivation and accelerate extracellular matrix degradation. Collectively, this “metabolic restoration” paradigm represents a conceptual advance over traditional “anti-activation” approaches and may ultimately enable the true reversal of established liver fibrosis.

## 10 Open questions and limitations

First, substantial cellular and stage-dependent heterogeneity of HSCs metabolism is under-appreciated. Most mechanistic experiments rely on fully activated HSCs populations *in vitro*, which poorly recapitulate the sequential *in vivo* metabolic shifts occurring across early-activation, full-activation, and partial-reversion states. Single-cell metabolomic datasets for human HSCs under different aetiologies (MASH, viral hepatitis, biliary injury) are still scarce, hindering the discovery and validation of stage-specific therapeutic nodes.

Second, the molecular machinery for erasing metabolic-epigenetic memory in HSCs remains largely obscure. Histone lactylation and persistent DNA-methylation marks establish pro-fibrotic epigenetic memory. Even after phenotypic reversal, some de-activated HSCs retain transcriptional priming and can be readily re-activated upon re-injury. The enzymes mediating histone delactylation in HSCs have not been systematically characterised, representing a major gap for achieving durable fibrosis resolution.

Third, target-cell-selectivity constitutes a major translational barrier. Key metabolic enzymes (GLS1, ACC, ACLY, HK2) are ubiquitously expressed across hepatocytes, macrophages and immune cells. Systemic small-molecule metabolic inhibitors frequently produce off-target effects. Delivery technologies that achieve HSCs-specific metabolic modulation (nanocarriers, cell-specific promoters) are still confined to pre-clinical studies and have not yet entered clinical pipelines.

Fourth, gut-liver-axis metabolite research faces a translational disconnect. Short-chain fatty acids, secondary bile acids and IPA show promising regulatory effects on HSCs plasticity in cell and animal experiments. Nevertheless, human data are limited; inter-individual microbiota variability makes it difficult to precisely tune hepatic metabolite concentrations. Whether dietary, probiotic or post-biotic interventions can stably drive HSCs phenotypic reversion in human patients awaits high-quality clinical evidence.

Fifth, clinical-trial endpoint design is sub-optimal. Current anti-fibrotic trials mostly rely on histopathological fibrosis-stage improvement as primary endpoints. Dynamic metabolomics and single-cell metabolic-flux readouts are rarely integrated as trial biomarkers. Therefore, it is difficult to judge whether investigational agents genuinely restore HSCs metabolic homeostasis or merely reduce hepatic inflammation and steatosis.

Sixth, existing literature predominantly focuses on suppressing pathological hyper-metabolism in activated HSCs, whereas the metabolic requirements sustaining long-term quiescent-state homeostasis of HSCs are insufficiently explored. Simple blockade of pro-activation pathways often fails to rebuild physiological metabolic balance, which partly explains why some metabolic inhibitors show robust efficacy in animal models but yield modest clinical outcomes.

Future investigations should combine single-cell metabolomics, dynamic metabolic-flux tracing, human liver organoid systems and humanised animal models to tackle these limitations, advancing therapeutic development from mere pathway inhibition toward genuine metabolic-homeostasis restoration.

## 11 Conclusion

In summary, metabolic reprogramming is central to hepatic stellate cell (HSCs) activation and liver fibrosis progression. Upon activation, HSCs exhibit coordinated shifts in glucose, lipid, and amino acid metabolism, including enhanced glycolysis with lactate-driven epigenetic regulation, lipid droplet degradation coupled with reduced fatty acid oxidation and increased lipogenesis, and altered metabolism of arginine, glutamine, proline, and branched-chain amino acids. These adaptations not only meet the energetic and biosynthetic demands of activated HSCs but also actively sustain their pro-fibrotic phenotype through metabolite-mediated signaling and epigenetics.

Crucially, these metabolic pathways represent actionable vulnerabilities rather than mere downstream consequences. Targeting key nodes—such as glycolytic enzymes, glutaminase, arginase, or lipid regulators—has shown promise in preclinical and early clinical studies, marking a shift from conventional anti-fibrotic strategies toward metabolic intervention. However, clinical translation faces challenges including target specificity, stage-dependent metabolic heterogeneity, and the need to restore, not simply block, metabolic homeostasis.

Future efforts should focus on defining stage-specific metabolic signatures, developing highly selective inhibitors, and integrating dynamic metabolomics into trial design. Only a comprehensive, metabolism-centric approach can achieve effective fibrosis reversal and improve long-term outcomes in chronic liver disease.
