# Supplementary material for: From metabolic antagonism to homeostatic restoration: rewiring hepatic stellate cell bioenergetics for liver fibrosis reversal
Source: Front Med (Lausanne). 2026 Sep 7;13:1912158. doi: 10.3389/fmed.2026.1912158 (PMC13548989; doi:10.3389/fmed.2026.1912158)
Supplement: Supplementary file 1 [file Table1.docx]

Table 1 Summary of anti-fibrotic therapeutic strategies targeting HSCs-related metabolic pathways

| **Therapeutic target** | **Mechanism of action** | **Representative compound** | **Targeted metabolic pathway** | **Research status** |
| --- | --- | --- | --- | --- |
| Glycolytic enzymes (HK2, GLUT1) | Suppress glycolysis, reduce lactate‑histone lactylation; block exosome‑mediated inter-HSCs crosstalk | Paeoniflorin-loaded Cu-doped ZIF-8 nanomaterial | Glucose metabolism | Pre-clinical^[86]^ |
| ACLY / ACSS2 | Block acetyl-CoA-dependent lipogenesis, repress HSCs activation | EVT0185 | Lipid metabolism | Clinical trial^[87]^ |
| Vitamin D receptor (VDR) | Down-regulate lipogenesis, up-regulate fatty-acid oxidation, restore PPARγ signalling | Natural VDR agonist from *Ligusticum chuanxiong* | Lipid metabolism | Pre-clinical^[88]^ |
| FGF21 signalling | Improve insulin sensitivity, enhance fatty-acid oxidation, inhibit HSCs pro-fibrotic phenotype | Efimosfermin alfa (BOS-580) | Lipid metabolism | Phase II clinical^[89]^ |
| DGAT2 | Inhibit triglyceride biosynthesis, mitigate lipotoxicity-driven HSCs activation | ION224 (antisense oligonucleotide) | Lipid metabolism | Phase II clinical^[90]^ |
| ROCK2 | Remodel perivascular microenvironment paracrine signals to indirectly restrain HSCs activation | TDI01 | No direct HSC-intrinsic metabolic target; microenvironment modulation | Clinical trial^[91]^ |
| GLS1 (glutaminase 1) | Attenuate glutaminolysis, lower α-KG abundance, curtail collagen precursor supply | Selective GLS1 small-molecule inhibitors | Amino-acid metabolism | Pre-clinical^[58,60]^ |
| ARG1 (arginase 1) | Block arginine-to-ornithine-proline flux, reduce collagen biosynthesis substrates | ARG1-selective inhibitors | Amino-acid metabolism | Pre-clinical^[54]^ |

Table 2 Summary of therapeutic strategies targeting hepatic stellate cell metabolism for liver fibrosis

| **Therapeutic agent/strategy** | **Target/pathway** | **HSCs relevance** | **Evidence level** | **Clinical stage** | **Major limitations** |
| --- | --- | --- | --- | --- | --- |
| **Systemic metabolic therapies for MASH (indirect modulation)** |  |  |  |  |  |
| Resmetirom | THR-β agonist | Indirect (improves hepatic lipid metabolism and systemic metabolic homeostasis) | Approved for MASH-related fibrosis | Marketed^81]^ | Mainly improves fibrosis, difficult to achieve complete reversal; lacks direct HSCs action |
| Tirzepatide | GLP-1R/GIPR dual agonist | Indirect (improves systemic metabolism and hepatic steatosis) | Positive phase 3 trial results | Phase 3^[82]^ | Common gastrointestinal adverse events; anti-fibrotic effects are secondary |
| Pegozafermin | FGF21 analogue | Indirect (improves systemic metabolism and insulin sensitivity) | Positive phase 2b trial results | Phase 2b^[83]^ | Common gastrointestinal adverse events |
| Survodutide | Glucagon/GLP-1R dual agonist | Indirect (improves systemic metabolism and hepatic steatosis) | Positive phase 2 trial results | Phase 2^[83]^ | Common gastrointestinal adverse events |
| **Indirect anti-fibrotic therapy** |  |  |  |  |  |
| Rifaximin-α (+ Lubiprostone) | Gut barrier / TLR4 signalling | Indirect (repairs intestinal barrier, reduces inflammatory signalling) | Preclinical and phase 2 trial | Phase 2^[84,85]^ | Only slows fibrosis progression; no evidence of reversal |
| **Direct anti-fibrotic therapies targeting HSC-intrinsic metabolism** |  |  |  |  |  |
| Nanomaterial (e.g., Cu-doped ZIF-8 loaded with paeoniflorin) | Key glycolytic enzymes (via Cu²⁺/Zn²⁺ release) | Direct (inhibits HSCs glycolysis and exosome secretion) | Preclinical (animal models) | Preclinical^[86]^ | Long-term safety and targeting efficiency of nanomaterials need validation |
| EVT0185 | Dual ACLY/ACSS2 inhibitor (acetyl-CoA homeostasis) | Direct (inhibits HSCs lipogenesis and activation) | Preclinical (human primary HSCs and mouse MASH models) | Phase 1^[87]^ | Early-stage; long-term efficacy and safety in humans unknown |
| VDR agonist (e.g., senkyunolide I) | VDR (regulates fatty acid metabolism) | Direct (reprogrammes HSCs fatty acid metabolism, inhibits synthesis and promotes oxidation) | Preclinical (cellular and animal models) | Preclinical^[88]^ | Requires more in vivo efficacy and safety data |
| FGF21 analogue (e.g., efimosfermin alfa / BOS-580) | FGF21 receptor (energy metabolism regulation) | Direct and indirect (directly inhibits HSCs activation, also improves systemic metabolism) | Positive phase 2b trial results | Phase 2b^[89]^ | Long-term follow-up data needed to confirm durability of fibrosis reversal |
| DGAT2 inhibitor (e.g., ION224) | DGAT2 (rate-limiting enzyme of triglyceride synthesis) | Direct (blocks lipotoxicity-induced HSCs activation) | Positive phase 2 trial results | Phase 2^[90]^ | Long-term effects of sustained lipogenesis inhibition on liver and other organs unclear |
| ROCK2 inhibitor (e.g., TDI01) | ROCK2 (perivascular microenvironment) | Indirect (suppresses HSCs activation by remodelling the hepatic sinusoid microenvironment) | Preclinical and early clinical | Phase 2^[91]^ | Not directly targeting HSCs metabolic enzymes; specific metabolic regulatory mechanisms remain to be clarified |

Figure 1 Metabolic remodeling of activated hepatic stellate cells (HSCs) drives liver fibrosis progression.

Figure legend：

Upon chronic liver injury, quiescent hepatic stellate cells (HSCs) become activated and undergo multi-layered metabolic reprogramming encompassing glucose, lipid, and amino-acid metabolic networks, which collectively act as key drivers of liver fibrosis progression.

In glucose metabolism, enhanced glycolysis generates lactate, which functions not merely as a metabolic by-product but as an epigenetic signal that triggers histone lactylation (e.g., H3K18la) to sustain persistent transcription of fibrogenic genes such as COL1A1 and α-SMA.

Lipid metabolism displays stage-dependent metabolic vulnerabilities: early-activated HSCs utilise lipophagy to break down lipid droplets and fuel fatty acid oxidation (FAO) for initial activation; fully-activated HSCs enter a unique dual hypermetabolic state with concurrent up-regulation of both glycolysis and tricarboxylic acid (TCA) cycle activity.

For amino-acid metabolism, the arginine‑proline pathway fuels collagen synthesis and extracellular-matrix (ECM) deposition; the glutaminase-driven glutamine pathway supplies substrates for the TCA cycle as well as glutathione (GSH) to support HSCs fibrogenesis, survival and oxidative-stress resistance.

From a translational perspective, therapeutic strategies targeting HSCs bioenergetics (including dual ACLY/ACSS2 inhibitor EVT0185, VDR agonists and ROCK2 inhibitors) aim to restore metabolic homeostasis rather than simply block HSCs activation, ultimately reversing the profibrotic phenotype. Future anti-fibrotic drug development should adopt stage-specific metabolic signatures, dynamic metabolomics-guided evaluation and highly selective metabolic modulators.

Figure 2. Metabolic reprogramming of glucose metabolism

Figure legend:

Quiescent hepatic stellate cells (HSCs) undergo pro-fibrotic activation upon liver injury, accompanied by comprehensive glucose metabolic reprogramming. Transforming growth factor-β (TGF-β) upregulates glucose transporter 1 (GLUT1) via canonical Smad2/3 and non-canonical p38 MAPK/PI3K/AKT pathways, and promotes GLUT1 membrane trafficking through the Src/SH3BP5/Rab11 complex. Hypoxia-inducible factor-1α (HIF-1α) synergistically enhances GLUT1 transcription. Activated HSCs secrete GLUT1-containing exosomes to trigger paracrine HSCs activation.

Activated HSCs adopt a Warburg-like aerobic glycolytic phenotype, with elevated hexokinase 2 (HK2), phosphofructokinase-1 (PFK1) and lactate dehydrogenase A (LDHA), and suppressed gluconeogenic enzymes. Excessive lactate induces histone lactylation to drive fibrotic gene expression. Lactate efflux via monocarboxylate transporters (MCT) causes extracellular acidification, which inhibits PHD2 and upregulates miR-182 to stabilize HIF-1α, forming a positive feedback loop that aggravates glycolysis and liver fibrosis progression.

Figure 3. Metabolic reprogramming of lipid metabolism

Figure legend:

Metabolic reprogramming of lipid metabolism during hepatic stellate cell (HSC) activation.In the healthy liver, quiescent HSCs maintain an anti-fibrotic phenotype characterized by abundant retinyl ester lipid droplets and high expression of key lipid metabolism regulators, including lecithin: retinol acyltransferase (LRAT), peroxisome proliferator-activated receptor γ (PPARγ), Adipor1, and C/EBPδ. Quiescent HSCs rely on fatty acid oxidation (FAO) for energy production. Upon liver injury and fibrotic stimulation, HSCs undergo activation. Lipid droplets are rapidly degraded via lipophagy, releasing free fatty acids (FFAs) that fuel early activation through autophagy/mitophagy, which also clears damaged mitochondria to sustain metabolic efficiency. Activated HSCs (myofibroblasts) exhibit profound metabolic reprogramming: enhanced aerobic glycolysis (the Warburg effect) and upregulated de novo lipogenesis (DNL), driven by acetyl-CoA carboxylase (ACC). Concurrently, LRAT is downregulated, while pro-fibrotic genes (α-SMA, type I collagen) are induced. Following injury cessation, some activated HSCs can undergo phenotypic reversal/inactivation via re-expression of transcription factors including PPARγ, GATA4, GATA6, and TCF21, leading to downregulated glycolysis and partial recovery of lipid storage, although transcriptomic differences from primitive quiescent HSCs persist.

Figure 4. Metabolic reprogramming of amino acid metabolism

Figure legend:

Activated hepatic stellate cells (HSCs) undergo comprehensive reprogramming of amino acid metabolism to support energy supply, redox homeostasis and extracellular matrix (ECM) synthesis. Arginine metabolism exhibits dual regulatory effects: nitric oxide synthase (NOS)-mediated arginine catabolism produces anti-fibrotic nitric oxide (NO), while upregulated arginase 1 (ARG1) redirects arginine flux toward ornithine, polyamines and proline synthesis, which serve as critical precursors for collagen deposition and promote liver fibrosis.

Enhanced glutaminolysis driven by glutaminase 1 (GLS1, activated via Hedgehog-YAP signaling) catalyzes glutamine degradation into glutamate and α-ketoglutarate (α-KG), which fuels the tricarboxylic acid (TCA) cycle, maintains glutathione (GSH)-dependent redox homeostasis and sustains HSC activation. Elevated serum glutamate/glutamine ratio correlates with fibrotic severity. Pyrroline-5-carboxylate reductase 1 (PYCR1) further mediates proline synthesis for ECM production.

Branched-chain amino acids (BCAAs) exert anti-fibrotic effects via mTORC1-dependent inhibition of TGF-β signaling; reduced Fischer ratio and BCAA/tyrosine ratio (BTR) are characteristic fibrotic biomarkers. Hepatocyte serine hydroxymethyltransferase 2 (SHMT2) and L-aspartate/mitochondrial axis also modulate hepatic inflammation and fibrotic progression.

**References**

86.	Zhang M, Jing H, Liu X, Milichko VA, Dou Y, Ren Y, et al. Intercellular communication interference through energy metabolism-related exosome secretion inhibition for liver fibrosis treatment. Acta Pharm Sin B. 2025;15(9):4900–16.

87.	Di Pastena F, Gautam J, Lally JSV, Fayyazi R, Grasset E, Bhattacharya D, et al. Dual inhibition of ACLY and ACSS2 by EVT0185 reduces steatosis, hepatic stellate cell activation, and fibrosis in mouse models of MASH. Cell Metab. 2026;38(1):33–49 e10.

88.	Zhu M, Ren L, Xiao W, Wang L, Hu Z, Wang N. Senkyunolide I suppresses hepatic stellate cell activation and liver fibrosis by reprogramming VDR-dependent fatty acid metabolism. Chin Med. 2025;20(1):85.

89.	Noureddin M, Kowdley KV, Odrljin T, Bain G, Zhao J, Jeglinski B, et al. Efimosfermin alfa (BOS-580) once per month in people with metabolic dysfunction-associated steatohepatitis with F2 or F3 fibrosis: results from a 24-week, randomised, double-blind, placebo-controlled, phase 2 trial. Lancet. 2026;407(10530):794–804.

90.	Loomba R, Morgan E, Yousefi K, Li D, Geary R, Bhanot S, et al. Antisense oligonucleotide DGAT-2 inhibitor, ION224, for metabolic dysfunction-associated steatohepatitis (ION224-CS2): results of a 51-week, multicentre, randomised, double-blind, placebo-controlled, phase 2 trial. Lancet. 2025;406(10505):821–31.

91.	Hu Y, Yang B, Xiao C, Yang X, Zhang H, Yang P, et al. Selective targeting of endothelial and perivascular angiocrine ROCK2 treats liver fibrosis. Cell. 2026.

58.	Du K, Chitneni SK, Suzuki A, Wang Y, Henao R, Hyun J, et al. Increased Glutaminolysis Marks Active Scarring in Nonalcoholic Steatohepatitis Progression. Cell Mol Gastroenterol Hepatol. 2020;10(1):1–21.

60.	Wang Y, Shi K, Tu J, Ke C, Chen N, Wang B, et al. Atractylenolide III Ameliorates Bile Duct Ligation-Induced Liver Fibrosis by Inhibiting the PI3K/AKT Pathway and Regulating Glutamine Metabolism. Molecules. 2023;28(14).

54.	Zhang M, Wu Z, Salas SS, Aguilar MM, Trillos-Almanza MC, Buist-Homan M, et al. Arginase 1 expression is increased during hepatic stellate cell activation and facilitates collagen synthesis. J Cell Biochem. 2023;124(6):808–17.

81.	Keam SJ. Resmetirom: First Approval. Drugs. 2024;84(6):729–35.

82.	Loomba R, Sanyal AJ, Kowdley KV, Bhatt DL, Alkhouri N, Frias JP, et al. Randomized, Controlled Trial of the FGF21 Analogue Pegozafermin in NASH. N Engl J Med. 2023;389(11):998–1008.

83.	Sanyal AJ, Bedossa P, Fraessdorf M, Neff GW, Lawitz E, Bugianesi E, et al. A Phase 2 Randomized Trial of Survodutide in MASH and Fibrosis. N Engl J Med. 2024;391(4):311–9.

84.	Israelsen M, Madsen BS, Torp N, Johansen S, Hansen CD, Detlefsen S, et al. Rifaximin-alpha for liver fibrosis in patients with alcohol-related liver disease (GALA-RIF): a randomised, double-blind, placebo-controlled, phase 2 trial. Lancet Gastroenterol Hepatol. 2023;8(6):523–32.

85.	Enomoto M, Kaji K, Nishimura N, Fujimoto Y, Murata K, Takeda S, et al. Rifaximin and lubiprostone mitigate liver fibrosis development by repairing gut barrier function in diet-induced rat steatohepatitis. Dig Liver Dis. 2022;54(10):1392–402.
